# LIGSITE^*csc*^: predicting ligand binding sites using the Connolly surface and degree of conservation

**DOI:** 10.1186/1472-6807-6-19

**Published:** 2006-09-24

**Authors:** Bingding Huang, Michael Schroeder

**Affiliations:** 1 Bioinformatics Group, Biotechnological Center, Technical University Dresden, Germany

## Abstract

**Background:**

Identifying pockets on protein surfaces is of great importance for many structure-based drug design applications and protein-ligand docking algorithms. Over the last ten years, many geometric methods for the prediction of ligand-binding sites have been developed.

**Results:**

We present LIGSITE^*csc*^, an extension and implementation of the LIGSITE algorithm. LIGSITE^*csc *^is based on the notion of surface-solvent-surface events and the degree of conservation of the involved surface residues. We compare our algorithm to four other approaches, LIGSITE, CAST, PASS, and SURFNET, and evaluate all on a dataset of 48 unbound/bound structures and 210 bound-structures. LIGSITE^*csc *^performs slightly better than the other tools and achieves a success rate of 71% and 75%, respectively.

**Conclusion:**

The use of the Connolly surface leads to slight improvements, the prediction re-ranking by conservation to significant improvements of the binding site predictions. A web server for LIGSITE^*csc *^and its source code is available at scoppi.biotec.tu-dresden.de/pocket.

## Background

In most cellular processes, proteins interact with other molecules to perform their biological functions. These interactions include the binding of ligands in receptor sites, the binding of antibodies to antigens, protein-DNA interactions, and protein-protein interactions. Shape complementarity has long been recognized as a major factor in these interactions [1-4]. The protein surface can form pockets, which are binding sites of small molecule ligands. The determination of pockets on protein surface is therefore a prerequisite for protein-ligand docking and an important step in structure-based drug design. In the last decade, many computational methods have been developed to predict and analyze protein-ligand binding sites. Many such as POCKET [5], LIGSITE [6], SURFNET [7], CAST [8], and PASS [9] use pure geometric characteristics and do not require any knowledge of the ligands.

**Figure 1:**
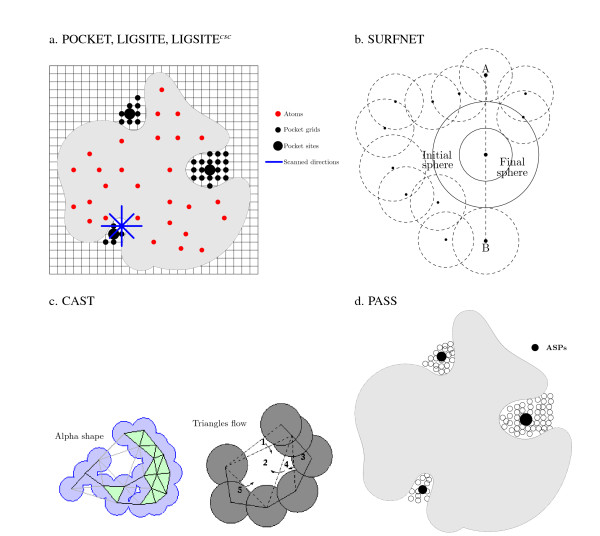
Pocket identification methods. **a**. POCKET, LIGSITE, and LIGSITE^*csc *^scan the grid for protein-solvent-protein and surface-solvent-surface events, respectively. POCKET uses 3, LIGSITE and LIGSITE^*csc *^7 directions. POCKET and LIGSITE use atom coordinates while LIGSITE^*csc *^uses the Connolly surface. **b**. SURFNET places a sphere, which must not contain any atoms, between two atoms. The spheres with maximal volume define the largest pocket. **c**. CAST triangulates the surface atoms and clusters triangles by merging small triangles to neighbouring large triangles. **d**. PASS coats the protein with probe spheres, selects probes with many atom contacts, and then repeats coating until no new probes are kept. The pockets, or active site points, are the probes with large number of atom contacts.

One of the first methods, POCKET [5], introduced the idea of protein-solvent-protein events as key concept for the identification (see Fig. 1a). The protein is mapped onto a 3D grid. A grid point is part of the protein if it is within 3 Å of an atom coordinate; otherwise it is solvent. Next, the *x*, *y*, and *z*-axes are scanned for pockets, which are characterized as a sequence of grid points, which start and end with the label protein and a period of solvent grid points in between. These sequences are called protein-solvent-protein events. Only grid points that exceed a threshold of protein-solvent-protein events are retained for the final pocket prediction. Since the definition of a pocket in POCKET is dependent on the angle of rotation of the protein relative to the axes, LIGSITE extends POCKET by scanning along the four cubic diagonals in addition to the *x*, *y *and *z *directions. LIGSITE was originally tested on 10 receptor-ligand complexes of which 7 bind in the largest, 2 in the second largest, and 1 in the third largest predicted pocket.

To further improve these results, we introduce two extensions to LIGSITE: First, instead of capturing protein-solvent-protein events, we capture the more accurate surface-solvent-surface events using the protein's Connolly surface [10], and not the protein's atoms. We call this extension LIGSITE^*cs *^(cs = Connolly surface). Second, we re-rank the pockets identified by the surface-solvent-surface events by the degree of conservation of the involved surface residues. We call this extension LIGSITE^*csc *^(csc = Connolly surface and conservation).

Three other approaches to pocket detection are SURFNET, CAST, and PASS. In SURFNET [7], the key idea is that a sphere, which separates two atoms and which does not contain any atoms, defines a pocket (see Fig. 1b). First, a sphere is placed so that the two given atoms are on opposite sides on the sphere's surface. If the sphere contains any other atoms, it is reduced in size until no more atoms are contained. Only spheres, which are between a radius of 1 to 4 Å are kept. The result of this procedure is a number of separate groups of interpenetrating spheres, called gap regions, both inside the protein and on its surface, which correspond to the protein's cavities and clefts. SURFNET was used to analyze 67 enzyme-ligand structures and the ligand is bound in the largest pockets in 83% of the cases [11].

CAST [8,12] computes a triangulation (see Fig. 1c) of the protein's surface atoms using alpha shapes [13,14]. In the next step, triangles are grouped by letting small triangle flow towards neighbouring larger triangles, which act as sinks. The pocket is then defined as collection of empty triangles. CAST was tested on 51 of 67 enzyme-ligand complexes used for SURFNET [11]. CAST achieves a success rate of 74%.

PASS [9] uses probe spheres to fill cavities layer by layer (see Fig. 1d). First, an initial coating of the protein with probe spheres is calculated. Each probe has a burial count, which counts the number of atoms within 8 Å distance. Only probes with count above a threshold are retained. This procedure is iterated until a layer produces no new buried probe spheres. Then each probe is assigned a probe weight, which is proportional to the number of probe spheres in the vicinity and the extent to which they are buried. Finally, a small number of active site points (ASP) are selected by identifying the central probes in regions that contain many spheres with high burial count. The final active site points are determined by cycling through the probes in descending order of probe weight, keeping only those above a threshold and farther than 8 Å apart from each other. Finally, the retained active site points are ranked by probe weight.

Besides the purely geometric methods above, there are methods, which take additional information into account to re-rank predictions. SURFNET's predictions were refined by considering the degree of residue conservation in the pocket [15]. Q-SITEFINDER [16] uses the interaction energy between the protein and a simple van Waals probe to locate energetically favorable binding sites.

The ultimate goal of ligand-binding sites prediction methods is to find active sites on uncharacterized structures. Therefore, it is of great importance to test and validate the methods on sufficiently large data sets. To this end, we use 210 bound structures from the Protein Ligand Database (PLD) [17] and 48 bound/unbound structures from [16] and [9].

## Implementation

### Algorithm

LIGSITE^*csc *^is an extension of LIGSITE. Instead of defining protein-solvent-protein events on the basis of atom coordinates, it uses the Connolly surface and defines surface-solvent-surface events. The algorithm proceeds as follows: First, the protein is projected onto a 3D grid. In order to minimize the necessary grid size, we apply principal component analysis so that the principal axis of the protein aligns with the *x*-axis, the second principal axis with the *y*-axis and the third with the *z*-axis. For the grid we use a step size of 1.0 Å. The rotation does not affect the quality of the results (data not shown), it only minimizes the necessary grid size. Second, grid points are labelled as *protein*, *surface*, or *solvent *using the following rules: A grid point is marked as *protein *if there is at least one atom within 1.6 Å. Next, the solvent excluded surface is calculated using the Connolly algorithm [10] and the surface vertices' coordinates are stored. In the Connolly algorithm, a hypothetical probe sphere (usual radius 1.4 Å) rolls over the protein. The Connolly surface is a combination of the van der Waals surface of the protein and the probe spheres surface, if the probe is in contact with more than one atom. A grid point is marked as *surface *if a surface vertex is within 1.0 Å. Note, that the distance thresholds ensure that all *surface *grid points are also labelled as *protein*. All other grid points are marked as *solvent*. Consider Fig. 1a. A sequence of grid points, which starts and ends with *surface *grid points and which has *solvent *grid points in between, is called a surface-solvent-surface event. LIGSITE^*csc *^scans the *x*, *y*, *z *directions and four cubic diagonals for such surface-solvent-surface events. If the number of surface-solvent-surface events of a *solvent *grid exceeds a minimal threshold (MINSSS, 6 in this work), then this grid is marked as *pocket*. Finally, all *pocket *grid points are clustered according to their spatial proximity. I.e. if a *pocket *grid point is within 3.0 Å to a *pocket *grid point cluster, it is added to this cluster. Otherwise, it becomes a new cluster. Next, the clusters are ranked by the number of grid points in the cluster. The top three clusters are retained and their centers of mass are used to represent the predicted pocket sites. This first extension to the basic LIGSITE algorithm is called LIGSITE^*cs*^. For LIGSITE^*csc*^, the top 3 pocket sites are re-ranked according to the degree of conservation of the involved surface residues. To be precise, the conservation score is the average conservation of all residues within a sphere of certain radius (8 Å here) of the center of mass of the cluster. The conservation score for each residue in a given protein is obtained from the ConSurf-HSSP database [18].

### LIGSITE, PASS, CAST, SURFNET implementations

In order to compare LIGSITE^*csc *^to LIGSITE, LIGSITE is implemented as well and the same parameters are used in both methods. A CAST pymol plugin was downloaded from cast.engr.uic.edu/cast/, PASS executable binaries (version 1.1) were requested from its authors and the SURFNET source code was obtained from .

### Datasets

To validate the binding site predictions we use two benchmark datasets of bound-only and bound/unbound structures. For the bound dataset, we use the Protein Ligand Database PLD [17], which is the largest hand-curated database containing all the protein-ligand complex structures available in the PDB. Currently, it has 485 protein ligand complexes (PLD v1.3). We removed redundant structures and selected those having conservation scores in the ConSurf-HSSP database (small peptides are not considered as ligand for 16 out of the 485 PLD structures). The result is a set of 210 structures (PDB codes are listed in the supplementary data table 6).

For a realistic evaluation, which takes into account flexibility of structures, we need bound and unbound structures. The predictions are made for the unbound structure and are checked against the bound structure. [19] presented a large test set of 305 ligand-bound protein complexes. Among these 305 structures, [16] created a data set of 35 structurally distinct proteins, for which there are also unbound structures. Additionally, [9] created a data set of 20 bound/unbound protein structures. The structure 2er6 is ignored since no ligand is found in the current PDB entry. Furthermore, there are five examples occurring in both data sets: 1stp, 2ypi, 1rbp, 1ifb, 3ptb and 5cpa. As a result, we have 48 bound/unbound structures on which we test LIGSITE^*csc*^, LIGSITE, PASS, CAST and SURFNET (see more details about 48 structures in the supplementary material, table 4 and 5).

**Figure 2:**
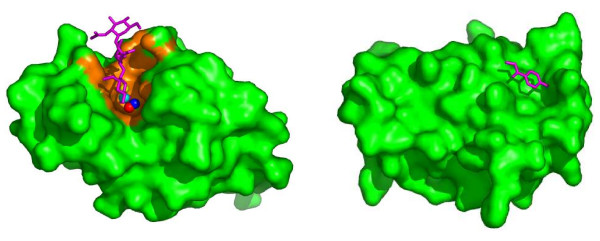
**Left**: Hen egg-white lysozyme with its ligand Tri-N-Acetylchitotriose (PDB 1hel). The ligand binds in a deep pocket and all algorithms correctly predict the binding site. red: LIGSITE^*csc*^, blue: LIGSITE, cyan: PASS, yellow: SURFNET, orange: CAST. **Right**: Hexameric insulin with its ligand methylparaben (PDB 6ins). The binding site of the ligand is unusually flat and therefore none of the methods detects it correctly.

In 28 (57%) cases, the five methods predict the same pockets as binding sites. Fig. 2 on the left shows such an example. These pocket sites are spatially similar and they are all the biggest pockets corresponding to the ligand binding sites. Fig. 2 on the right shows a case where all methods fail, since the binding site is nearly flat, so that the assumption that the ligand binds at a large pocket, does not hold.

To further validate the algorithms, LIGSITE^*csc*^, LIGSITE, SURFNET, and PASS are tested on non-redundant bound structures of 210 protein-ligand complexes from the Protein Ligand Database. CAST is not evaluated since we only get a Pymol plugin for it, which has to be used manually. As summarized in Table 2, LIGSITE^*csc *^performs slightly better than the others and achieves an overall success rate of 75% for top 1 predictions.

The predicted pocket sites are classified into four classes following [11]: the ligand binding sites is the first, second, third largest pocket or none of these. Table 3 shows the percentage for these four classes, as well as the average and the stadard deviation of the size of the pocket sizes in term of the number of *pocket *grid points. The goal of re-ranking by conservation is to bring hits found in the second and third largest pocket to rank 1. The ratio of the largest pocket to the second largest for a given protein approximately indicates how unusually large the largest pocket is. For binding sites in the largest pocket the ratio is greater than for binding sites in the second and third largest pocket. To put it differently, if the largest pocket is significantly larger than the others, then it is likely the binding site, otherwise the other two pockets are likely, too. There are 27 cases in the fourth class that the ligand does not bind to any of the top 3 pocket sites (see Table 3). Among these 27 structures, there are 11 cases that the ligan-binding site is around a small pocket and the ranking of this site in LIGSITE^*cs *^is behind 3. Ligsitecs fails to identify binding sites for the other 16 structures. However, among these 16 cases there are 12 proteins that the ligand-binding site is near the biggest pocket. LIGSITE^*cs *^can identify these pocket sites at the top 1 if the distance threshold is set to 8.5 *Å*. The ligand-binding site is geometrical flat for only 4 cases (1ac0,1l82,1rgk and 2msb). However, the binding site is more conserved than the rest of the surface except for 1182 in these 4 cases. None of the geometrical methods can detect such flat binding sites.

The structures are prepared as follows: All solvent molecules including phosphate, sulphate and metal ions are ignored in the unbound structures. Next, the bound and unbound structures are aligned using PyMol [20]. Note, that the choice of structural alignment algorithm is not significant, as nearly identical structures are aligned, which only differ in some conformational changes. After each tool predicts ligand binding sites the predictions have to be rated. This is a difficult task as the methods follow different approaches and use different evaluation methods. For example, [6] measure the accuracy by the percentage of predicted pocket atoms that are in contact with the ligand. A protein and ligand atom are in contact if they are within a distance of the sum of the van der Waals radii plus 0.5 Å [16] used a precision threshold for success in which at least 25% of probe sites in a single cluster are within 1.6 Å to a ligand atom. Alternatively, the success rate of predictions can be measured by computing the distance between the ligand and a single point representing the pocket [9]. To assess different methods on the same data set, we need a common criterion for success. Therefore, we take a distance-based approach. For LIGSITE^*csc *^and LIGSITE, this point is the geometric center of the pocket sites' grid points. In PASS, the pockets are represented by its active site point ranked by their probe weight. In SURFNET, the default "gaps.pdb" output file is a PDB-format file in which each gap region generated by SURFNET is represented by a single ATOM record. Each atom is located at the center of mass position of the corresponding gap region, and the atoms can be used to represent the predicted pocket sites ranked by their volume. CAST defines atoms belonging to a pocket. The pocket can be represented by its center of mass. Thus, for all methods we can define a single point which represents the predicted pocket and we can compute the distance of this point from the ligand. A prediction is a hit if it is within 4 Å to any atom of the ligand.

### Negative datasets

Evaluating protein interactions is inherently difficult, as not all interactions are known. A positive dataset of true interactions as defined above cannot be assumed to be complete. Negative datasets of experimentally confirmed non-interactions are not available [21]. Therefore, researchers working on protein-protein interactions infer non-interactions from randomly selected pairs of proteins. Such pairs are apriori unlikely – but not impossible – to interact. The likelihood that they do interact is low, as there is a quadratic number of pairs of proteins, while the number of truly interacting proteins is comparatively low. [22] estimate e.g. only 10.000 types of interactions in the light of an estimated 1000 structural folds. A second approach to infer negative datasets, additionally requires that the protein pairs are in different cellular locations [23]. This additional requirement indeed ensures that they cannot possibly interact. While improving the quality of the data, this additional requirement introduces a bias in the negative dataset, as the protein pairs in different cellular locations are not representative of all pairs [21].

To summarise, the definition of negative interaction datasets is difficult, but we can follow a similar approach for protein-ligand interactions as done for protein-protein interactions. We define two negative datasets: The first consists of 1000 randomly selected surface patches. These patches are apriori unlikely – but not impossible – ligand binding sites. Here, a surface patch consists of a randomly selected surface exposed C_*α *_and all C_*α *_atoms with 8 Å and 10 Å, respectively. For comparison, the area of a circle of these radii is 800 Å and 1300 Å and the volume of a sphere of these radii is 2100 Å and 4200 Å, respectively. These values give a broad comparison to protein interface sizes ranging from small ones less than 600 Å to large ones greater than 2000 Å.

The second negative dataset consists of 1000 randomly selected hetero permanent protein-protein interaction interfaces. As the interface is used by a protein complexe it cannot be a ligand binding site. The permanent interactions were selected from the SCOPPI database [24,25].

To determine whether our method predicts any of these negative surface patches, we consider a predicted ligand binding site to hit a surface patch, if at least 50% of the residues overlap. Before we discuss the results of LIGSITE^*csc *^on these negative datasets, we will discuss the results for all methods on the positive dataset.

## Results and discussion

Table 1 shows the success rates using these five methods on 19 complexes from PASS [9] and 29 complexes from [16], excluding those structures already existing in PASS, for unbound and bound structures. For unbound structures, LIGSITE^*cs *^achieves both for the top prediction and the top three predictions the best overall success rates. Using the geometric feature alone, LIGSITE^*cs *^can identify ligand-binding sites at 60% and 77% accuracy for the top 1 and top 3 pocket sites, respectively. In the second stage of re-ranking by conservation, LIGSITE^*csc *^correctly re-ranks 34 out 37 top 3 predictions by LIGSITE^*cs*^. Thus, LIGSITE^*csc *^improves the success rate of top 1 predictions from 60% to 71%. For bound structures results are generally better (see Table 1). For the bound structures, LIGSITE^*csc *^improves the success rate from 69% to 79% for the first prediction. These results indicate that conformational changes pose a challenge for all methods. In 2tga/1mtw and 3gch/1chg, the loops near the ligand binding sites stretch significantly to allow ligand binding. None of the methods predicts the site correctly. However, this ligand binding sites is the biggest pocket on bound structure and is highly conserved (data not shown).

**Figure 3:**
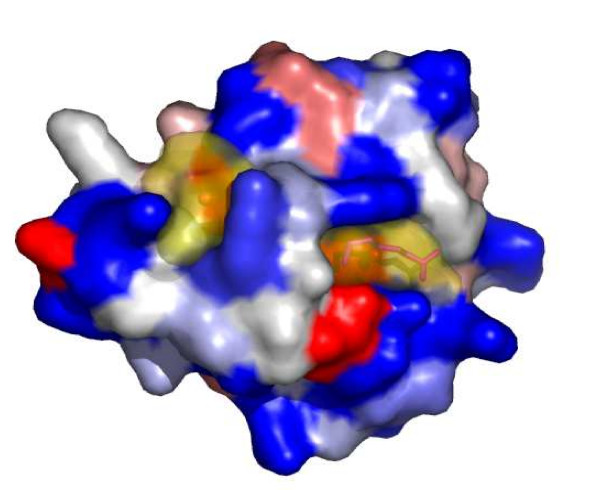
Mapping pockets and degree of conservation onto a protein surface (1krn). The first two pockets have similar size (ratio: 1.3). The residue near the second largest pocket (right, yellow), which is the ligand binding site, are more conserved than those near the largest pocket (left, yellow). Red: highly conserved, grey: less conserved.

Conservation has been widely used for function site prediction [26-28] and protein-protein interaction interface prediction [29-32], combined with other physiochemical properties. Here, we propose to re-rank the top 3 geometric-based prediction using the degree of conservation of the involved residues. As a result, we can improve the ranking for 183 out of 210 structures, which are hits of LIGSITE^*cs*^'s top 3 predictions. LIGSITE^*csc *^correctly ranks 157 out of these 183 as top 1 (86%). Fig. 3 shows a typical example that how conservation score improves the ranking for a Kringle domain (pdbid 1krn).

**Figure 4:**
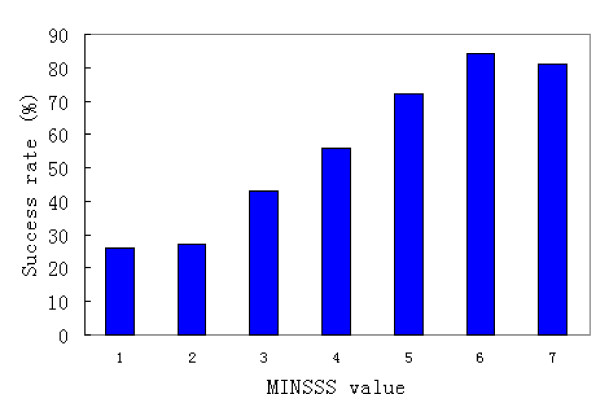
The success rates of LIGSITE^*cs *^for different thresholds for the minimal number of surface-solvent-surface events, MINSSS, for top 3 predictions for 210 bound structures.

**Figure 5:**
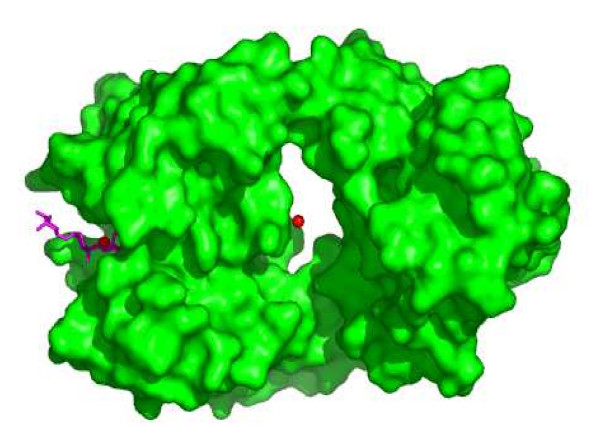
Limits of LIGSITE^*csc*^: The hole in a ring structure (pdbid 1a4j) is predicted by LIGSITE^*csc *^as largest pocket. The ligand binds, however, to the second largest pocket shown on the left.

In LIGSITE^*csc*^, there are four key parameters which influence the results, namely grid size, minimal number of surface-solvent-surface events (MINSSS), the radius of the sphere to calculate the conservation score and the distance threshold for defining hits (see Methods and Materials). For grid size, we tested LIGSITE^*csc *^using 0.8, 0.9, 1.1 and 1.2 Å. The success rates only vary -5 to +5 percentage for the 210 bound structures (data not shown). Although a smaller grid size leads to finer-grained pockets, the ranking is not affected. Additionally, smaller grids leads to cubically increasing run-time. Thus we choose 1.0 Å. The surface-solvent-surface events (protein-solvent-protein events in LIGSITE) vary from 1 (buried) to 7 (very deeply buried). Fig. 4 shows the success rates of LIGSITE^*cs *^for different MINSSS values on the 210 bound structures. The cutoff of 6 leads to the best results and is therefore chosen. Scanning along Nonetheless, scanning along 7 directions fails if the structure forms a ring (see Fig. 5). As mentioned earlier, at the second stage, the top 3 pocket sites are re-ranked by the average conservation score of residues with a sphere of radius 8 Å. This radius ensures a moderate size of patch within this sphere, which gives a reasonable average conservation score for re-ranking.

**Figure 6:**
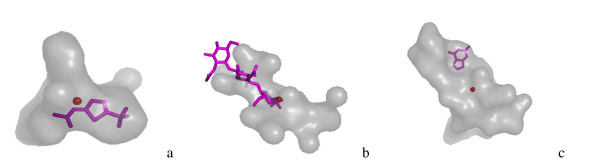
The occupancy of ligands on predicted pocket sites. Grey: the whole pocket sites, Red: mass center of pocket sites and Magenta: ligand. **a**). Carbonic anhydrase II (2cba), a perfect prediction. **b**). Acetylchitotriose (1hel) good prediction but only a small part of ligand atoms occupy the pocket sites. **c**). Purine nucleoside phosphorylase (1ula), the pocket sites cover all atoms of the ligand. The minimal distance is 5.10 Å since ligand is very small and it is not counted as a hit.

Representing the pocket site as the mass center of grid clusters is somehow too simple for very large pockets. The ligand does not occupy the whole pocket sites and does not locate around the center of the pocket sites. Also, the orientation of ligand and the shape of the pocket sites are very important for the assessment of predictions. Fig. 6a shows a perfect prediction on Carbonic anhydrase II (pdbcode 2cba). In this case, the pocket sites cover all ligand atoms and the minimal distance between the mass center of this pocket and the ligand is 1.8 Å. However, as shown in Fig. 6b, on Acetylchitotriose (pdbcode 1hel), only a small part of ligand atoms occupy the pocket sites. In Fig. 6c, the ligand is very small comparing to the pocket site it locates on Purine nucleoside phosphorylase (pdbcode 1ula). The minimal distance between them is 5.10 Å, which is not counted as a hit (4 Å is used to define a hit). This phenomenon might be a reason why the success rates of SURFNET here are lower than reported in [11], which used a different hit definition. However, increasing the distance threshold does not improve the performance of LIGSITE^*csc *^significantly (data not shown). Nevertheless, the advantage of representing pockets as a single point is that different methods can be assessed by the same criteria. Moreover, rather than using the original grid points in the cluster, it is straightforward to extend this single point using a sphere of a certain radius.

Finally, let us consider LIGSITE^*csc*^'s performance on the negative datasets. As described in the implementation section, we defined two negative datasets of surface patches, which are unlikely binding sites and hence serve as a negative control. I.e. LIGSITE^*csc *^should not predict any of these sites as possible ligand binding sites. The first set consists of 1000 randomly selected surface patches, for which we varied the radius between 8 and 10 Å. LIGSITE^*csc *^misclassifies 8% (8 Å radius surface patch) and 23% (10 Å radius). The range from 8% to 23% is not surprising as the volume of a sphere doubles as its radius changes from 8 to 10 Å.

The second negative dataset consists of 1000 permanent protein complex interfaces. LIGSITE^*csc *^misclassifies 13% as predicted ligand binding sites. These results are in line with [33], who analysed pockets involved in protein-protein and protein-ligand interactions and found that there are fundamental differences including conservation. Thus, LIGSITE^*csc *^achieve reasonable results on the negative controls, further strengthening the positive results discussed above.

## Conclusion

In the last decade, many computational methods have been developed to identify pockets on protein surfaces and to analyze the relationship between the pockets and ligand-binding sites. Most of them are purely geometric and do not require any knowledge of the ligands. However, there is no comparison between these methods. In this paper, we propose a method called LIGSITE^*csc*^, which extends LIGSITE [6] by defining surface-solvent-surface events and ranking them by the degree of conservation [15]. We compare LIGSITE^*csc *^to LIGSITE, PASS, SURFNET, and CAST on a dataset of 48 unbound/bound and 210 bound-only protein-ligand complexes using the same evaluation criteria. On the unbound/bound complexes, the methods predict the same correct ligand-binding sites in 28 out of 48 cases. Overall, LIGSITE^*csc *^performs slightly better than the other approaches and correctly predicts the ligand binding site in 71% and 75% cases, respectively.

## Availability and requirements

LIGSITE^*csc *^is online at scoppi.biotec.tu-dresden.de/pocket. Users can submit PDB files or enter a PDB ID and specify the chain ID. The parameters can be adjusted by the user. It returns the pocket sites in a standard PDB file format and a python script for visualization of pockets using PyMol [20] as well. LIGSITE^*csc *^and LIGSITE are both implemented in C++ using the BALL [34] library. LIGSITE^*csc*^'s C++ source code is freely available for academic users from the web site, and as additional file 1 in compliment to this manuscript.

## Authors' contributions

BH carried out the research and drafted the manuscript. MS guided the research and revised the manuscript.

## Figures and Tables

**Table 1:** Success rates for 48 unbound/bound structures (percentage).

Method	Top 1		Top 3	
	
	unbound	bound	unbound	bound
LIGSITE^*csc*^	71	79		
LIGSITE^*cs*^	60	69	77	87
LIGSITE	58	69	75	87
CAST	58	67	75	83
PASS	60	63	71	81
SURFNET	52	54	75	78

**Table 2:** Success rates for 210 bound structures.

Method	Top1	Top3
LIGSITE^*csc*^	75%	
LIGSITE^*cs*^	67%	87%
LIGSITE	65%	85%
PASS	54%	79%
SURFNET	42%	56%

**Table 3:** Numbers of protein in each class for 210 bound structures.

Class	No. of proteins (as %)	Avg no. pocket points	Stdev
Class 1: Binding site in largest pocket	141/210 = 67%	209	185
Class 2: Binding site in second largest pocket	28/210 = 13%	66	64
Class 3: Binding site in third largest pocket	14/210 = 7%	40	41
Class 4: Binding site in none of above	27/210 = 13%		

**Table 4:** Comparison of LIGSITE^*csc*^, LIGSITE, PASS, SURFNET, CAST on 48 unbound structures.

Complex	Unbound	LIGSITE^*csc *1^		LIGSITE^2^		PASS^3^		SURFNET^4^		CAST	
		
	PDB	Hits^5^	*D*_*Near*_^6^	Hits	*D*_*Near*_	Hits	*D*_*Near*_	Hits^7^	*D*_*Near*_	Hits	*D*_*Near*_
1bid	3tms	1	3.4	1	2.0	1	3.9	1	3.9	1	3.1
1cdo	8adh	1	0.8	1	0.6	1	0.2	1	1.3	1	0.8
1dwd	1hxf	1	1.7	1	2.3	1	0.7	1	2.3	1	0.9
1fbp	2fbp	1	0.5	1	0.6	(2)	0.8	-	-	1	1.5
1gca	1gcg	1	0.8	1	0.8	1	0.5	1	3.4	1	0.5
1hew	1hel	1	1.8	1	1.8	1	1.0	1	2.6	1	1.6
1hyt	1npc	1	1.2	1	1.1	1	1.7	1	1.0	1	0.7
1inc	1esa	1	2.9	3	0.8	-	-	1	1.9	(10)	2.1
1rbp	1brq	1	0.9	1	0.9	1	0.9	(2)	1.6	1	1.0
1rob	8rat	1	0.9	2	1.0	1	0.3	1	1.7	1	1.6
1stp	1swb	1	0.6	1	0.3	1	0.8	1	2.4	1	1.4
1ulb	1ula	-	-	(20)	3.2	-	-	1	3.6	1	3.3
2ifb	1ifb	1	2.2	1	2.2	1	2.5	1	2.3	1	2.1
3ptb	3ptn	1/2	1.1	2	1.0	(2)	0.5	(2)	1.7	1	0.9
2ypi	1ypi	-	3.0	2	3.0	(3)	2.2	-	-	(2)	2.7
4dfr	5dfr	1	1.9	1	3.5	1	2.3	-	-	1	4.5
4phv	3phv	1	2.7	1	2.6	-	-	1	2.9	1	2.6
5cna	2ctv	1/11	1.0	(13)	1.0	(2)	0.8	(6)	1.1	(6)	1.0
7cpa	5cpa	1	1.0	1	1.1	1	1.3	1	1.6	(3)	1.0
											
1a6w	1a6u	1/3	0.5	(4)	1.4	-	-	-	-	1	1.4
1acj	1qif	-	3.5	-	3.6	1	1.9	-	-	(40)	3.9
1apu	3app	-	1.2	-	1.9	-	-	1	3.7	-(2)	-(4.1)
1blh	1djb	1	0.7	2	1.2	1	2.4	(2)	3.9	(5)	0.8
1byb	1bya	1	2.5	1	2.8	(4)	1.1	-1	-(4.2)	1	2.4
1hfc	1cge	1	0.7	1	0.9	(3)	0.8	(3)	1.2	1	0.5
1ida	1hsi	1	3.4	1	2.9	(3)	1.0	1	1.0	1	1.6
1igj	1a4j	/4	0.8	-(19)	2.9	-	-	-	-	-	-
1imb	1ime	1	1.7	1	1.0	1	1.7	1	4.0	1	1.3
1ivd	1nna	1	1.4	1	1.1	1	3.5	(2)	0.9	1	1.9
1mrg	1ahc	1	1.9	1	1.9	-	-	1	3.3	1	0.8
1mtw	2tga	1/5	2.8	-(7)	1.2	-	-	(7)	3.2	(8)	1.6
1okm	4ca2	1	2.2	1	1.6	-	-	(3)	2.2	1	2.1
1pdz	1pdy	1	2.6	1	3.1	1	1.7	-	-	(5)	1.0
1phd	1phc	1	0.7	1	1.2	1	1.8	(2)	1.4	1	1.3
1pso	1psn	1	0.8	1	1.6	1	1.6	-1	-(4.3)	1	2.1
1qpe	3lck	2	1.5	2	1.2	1	0.7	-	-	-	-
1rne	1bbs	1	1.0	1	1.2	1	1.4	1	2.2	1	1.0
1snc	1stn	1	1.5	1	1.5	1	1.3	1	1.9	1	1.3
1srf	1pts	1	1.5	1	0.5	1	1.2	(5)	0.8	1	1.1
2ctc	2ctb	1	0.6	1	1.1	(2)	0.8	1	2.2	1	1.2
2h4n	2cba	1/2	1.0	2	1.0	-	-	(3)	1.2	(2)	1.2
2pk4	1krn	1/2	0.7	2	0.8	-	-	(2)	2.2	1	1.9
2sim	2sil	1/2	0.7	2	0.6	-	-	(2)	2.3	(2)	0.8
2tmn	1l3f	-	2.1	-	-	-	-	1	0.7	1	3.9
3gch	1chg	10	2.2	-(10)	2.2	1	0.9	(11)	1.5	(2)	2.5
3mth	6ins	9	3.8	-(9)	1.8	-	-	-(3)	-(4.7)	-	-
5p2p	3p2p	1	1.3	1	1.6	1	1.8	(2)	1.6	(2)	1.5
6rsa	7rat	1/4	0.9	-(5)	1.1	1	1.1	1	0.6	1	0.9

^1^Grid resolution: 1.0 Å; probe radius: 1.6 Å.

^2^Parameters are the same as LIGSITE^*csc*^.

^3^The values are directly taken from PASS [9]. Only the best hit is shown.

^4^Grid separation: 1.0 Å. Minimum and maximum radius for gap spheres: 1.0 and 4.0 Å. The "gaps.pdb" file is used for representation for pocket sites.

^5^Hits: PS(s) lying within 4 Å of the superimposed ligand. Only the best hit is shown. A dash indicates that no hit is found, brackets indicate hits, which are no top hits.

^6^Distances from hits to the nearest atom of superimposed ligand, unit: Å.

^7^PS(s) lying within 4 Å of the superimposed ligand.

**Table 5:** Overview of the data set of 48 bound/unbound structures.

Complex	Unbound	RMSD (Å)^1^	Protein Description	Ligand Description^2^
1bid	3tms	0.24	Thymidylate synthase	CBX, UMP
1cdo	8adh	1.17	Alcohol dehydrogenase	NAD
1dwd	1hxf	0.44	Alpha thrombin + hirudin	MID
1fbp	2fbp	0.89	Phosphohydrolase	AMP, F6P
1gca	1gcg	0.32	Galactose-binding protein	GAL
1hew	1hel	0.21	Acetylchitotriose	NAG
1hyt	1npc	0.87	Thermolysin	DMS, BZS
1inc	1esa	0.21	Elastase	ICL
1rbp	1brq	0.54	Retinol binding protein	RTL
1rob	8rat	0.28	Ribonuclease A	C2P
1stp	1swb	0.33	Streptavidin	BTN
1ulb	1ula	0.61	Purine nucleoside phosphorylase	GUN
2ifb	1ifb	0.37	Fatty acid binding protein	PLM
3ptb	3ptn	0.26	Beta trypsin	BEN
2ypi	1ypi	0.57	Triose phosphate isomerase	PGA
4dfr	5dfr	0.80	Dihydrofolate reductase	MTX
4phv	3phv	1.28	HIV 1 protease	VAC
5cna	2ctv	0.44	Concanavalin A	MMA
7cpa	8adh	2.17	Carboxypeptidase	FVF
1a6w	1a6u	0.35	B1-8 FV fragment	NIP
1apu	3app	0.36	Penicillopepsin	MAN, OET, IVA, STA
1acj	1qif	0.34	Acetylcholinesterase	THA
1blh	1djb	0.23	Methyl]phosphonate	FOS
1byb	1bya	0.26	Beta amylase	GLC
1hfc	1cge	0.37	Fibroblast collagenase	HAP
1ida	1hsi	1.41	HIV 2 protease	QND, HPB, PY2, PPL
1ivd	1nna	1.00	Sialidase	FUC, ST1, NAG, MAN
1mrg	1ahc	0.30	Alpha momorcharin	AND
1mtw	2tga	0.31	Trypsin	DX9
1okm	4ca2	0.34	carbonic anhydrase II	SAB
1pdz	1pdy	0.54	Enolase	PGA
1phd	1phc	0.17	Camphor 5-monoxygenase	HEM, PIM
1pso	1psn	0.33	Pepsin 3a	IVA, STA
1qpe	3lck	0.25	Lck kinase	PP2, PTR
1rne	1bbs	0.60	Renin	NAG, C60
1snc	1stn	0.52	Staphylococcal nuclease	PTP
1srf	1pts	0.45	Streptavidin	MTB
1stp	2rta	0.62	Streptavidin	BTN
2ctc	2ctb	0.15	Carboxypeptidase	LOF
2h4n	2cba	0.33	Carbonic anhydrase II	AZM
2pk4	1krn	0.63	Plasminogen kringle	ACA
2sim	2sil	0.25	Sialidase (neuraminidase)	DAN
2tmn	1l3f	0.62	Thermolysin	PHO, NH2
3gch	1chg	0.91	Gamma chymotrypsin	CIN
3mth	6ins	1.00	Methylparaben insulin	MPB
5p2p	3p2p	0.62	Phosphilipase	DHG
1imb	1ime	1.45	Inositol monophosphatase	LIP
6rsa	7rat	2.08	Ribonuclease	UVC

^1^RMSD: Root mean square deviation of C*α *atoms after superimposing unbound structures on bound structures.

^2^There letters abbreviation in PDB, separated by "," if more than one

**Table 6:** The PDB code of 210 protein-ligand complexes taken from the PLD database.

1a0q	1a28	1a42	1a4g	1a6w	1a9u	1aaq	1abe	1ac0	1acj	1aco	1adb
1add	1adf	1aec	1aha	1ai5	1aj7	1ake	1anf	1aoe	1apt	1ase	1azm
1b59	1b6n	1b9v	1baf	1bap	1bcd	1bgo	1bhf	1bl7	1blh	1bma	1bmq
1bra	1byb	1byg	1c2t	1c5c	1c5x	1c83	1cbs	1cbx	1cdg	1ckp	1cla
1cle	1coy	1cps	1cqp	1ctr	1ctt	1d0l	1d3h	1dbb	1dd7	1dg5	1dhf
1did	1dih	1dmp	1dog	1dr1	1e96	1eap	1ebg	1eed	1ei1	1ejn	1ela
1eoc	1epb	1eta	1exw	1f0r	1fbl	1fen	1fgi	1fkb	1fki	1fmo	1frp
1glp	1gpy	1hak	1hbv	1hdy	1hew	1hfc	1hti	1hyt	1ibg	1icn	1ida
1imb	1inc	1ivb	1ivc	1jao	1l82	1lah	1lcp	1ldm	1lgr	1lic	1lmo
1lpm	1mbi	1mfc	1mmp	1mmq	1mrg	1mrk	1mts	1mup	1nco	1nsc	1okl
1pbd	1pdz	1pgp	1pha	1poc	1ppi	1ppk	1pso	1qbr	1qcf	1qh7	1qpe
1rbp	1rds	1rgk	1rne	1rob	1rpa	1rt2	1sln	1slt	1snc	1sre	1stp
1tdb	1thl	1tlc	1tng	1tph	1ukz	1ulb	1uvs	1vgc	1xid	1ydr	2aad
2ack	2ada	2ak3	2cmd	2cpp	2csc	2ctc	2er0	2fox	2gbp	2gpb	2ifb
2msb	2phh	2pk4	2qwb	2sim	2sns	2tsc	2xis	2yhx	2ypi	3cla	3dfr
3er3	3ert	3fx2	3gch	3gpb	3hvt	3nos	3ts1	4cts	4dfr	4est	4gr1
4hvp	4lbd	4mbp	4tln	4xia	5abp	5cpp	5er1	5p21	5p2p	6acn	6cpa
6rnt	6rsa	7lpr	7tim	9aat	9icd						
